# YOLOv10n-CF-Lite: A Method for Individual Face Recognition of Hu Sheep Based on Automated Annotation and Transfer Learning

**DOI:** 10.3390/ani15172499

**Published:** 2025-08-25

**Authors:** Yameng Qiao, Wenzheng Liu, Fanzhen Wang, Hang Zhang, Jinghan Cai, Huaigang He, Tonghai Liu, Xue Yang

**Affiliations:** 1 College of Computer and Information Engineering, Tianjin Agricultural University, Tianjin 300392, China; qym18822041085@126.com (Y.Q.); wenzheng_0307@163.com (W.L.); wangfanzhen@tjau.edu.cn (F.W.); zhangh@tjau.edu.cn (H.Z.); q1463747994@163.com (J.C.); 2 Qingyang Academy of Agricultural Sciences, Qingyang 745000, China; huoqiusi@163.com; 3 College of Engineering and Technology, Tianjin Agricultural University, Tianjin 300392, China

**Keywords:** individual recognition, automatic annotation, YOLOv10, transfer learning

## Abstract

Individual recognition of Hu sheep is a core requirement for precision livestock management, significantly improving breeding efficiency and fine management. However, traditional machine vision methods face challenges such as high annotation time costs, the inability to quickly annotate new sheep, and the need for manual intervention and retraining. To address these issues, this study proposes a solution that integrates automatic annotation and transfer learning, developing a sheep face recognition algorithm that adapts to complex farming environments and can quickly learn the characteristics of new Hu sheep individuals. First, through multi-view video collection and data augmentation, a dataset consisting of 82 Hu sheep and a total of 6055 images was created. Additionally, a sheep face detection and automatic annotation algorithm was designed, reducing the annotation time per image to 0.014 min compared to traditional manual annotation. Next, the YOLOv10n-CF-Lite model is proposed, which improved the recognition precision of Hu sheep faces to 92.3%, and the mAP@0.5 to 96.2%. To enhance the model’s adaptability and generalization ability for new sheep, transfer learning was applied to transfer the YOLOv10n-CF-Lite model trained on the source domain (82 Hu sheep) to the target domain (10 new Hu sheep). The recognition precision in the target domain increased from 91.2% to 94.9%, and the mAP@0.5 improved from 96.3% to 97%. Additionally, the model’s convergence speed was improved, reducing the number of training epochs required for fitting from 43 to 14. In summary, the Hu sheep face recognition algorithm proposed in this study improves annotation efficiency, recognition precision, and convergence speed through automatic annotation and transfer learning. It can quickly adapt to the characteristics of new sheep individuals, providing an efficient and reliable technical solution for the intelligent management of livestock.

## Simple Summary

Individual recognition of Hu sheep is a core requirement in precision livestock management. However, traditional machine vision methods face several challenges in practical applications, such as high annotation time costs, the inability to quickly annotate new sheep, and poor adaptability to new individuals. To address these issues, this study proposes an innovative solution that integrates automatic annotation and transfer learning technologies. The automatic annotation not only significantly reduces the workload when new sheep are introduced into the farm but also effectively improves the model’s adaptability and generalization ability to new sheep. Meanwhile, the YOLOv10n-CF-Lite model ensures a lightweight design while still providing high-precision recognition of Hu sheep, further optimizing the individual recognition process.

## 1. Introduction

With the acceleration of modernization, digitalization, and intelligence in animal husbandry, precision farming has gradually become the focus of the industry. In this context, building a digitalized farm closely integrated with big data has become particularly important, especially in collecting individual information of animals such as Hu sheep, which has become the core of modern farm management [1]. By accurately identifying each Hu sheep’s identity, it is possible not only to effectively prevent diseases and collect individual data in real-time but also to help develop scientific breeding strategies, improve breeding efficiency, optimize feeding methods, and reduce farming costs [2]. Furthermore, animal identity recognition ensures the traceability of meat product quality, meeting consumers’ demand for high-quality meat, thus promoting precision animal husbandry towards a more efficient and intelligent direction.

Currently, domestic livestock farms primarily rely on RFID and computer vision technology for individual animal identification. RFID technology, with its high efficiency and accuracy, allows for rapid animal identification and stable operation in various environmental conditions, offering strong adaptability. However, RFID tags have certain limitations, as frequent animal activity, harsh environmental conditions, improper installation, and the quality of the tags themselves can lead to tag detachment or damage, thereby affecting the accuracy of animal identification and the continuity of data. In addition, RFID technology typically has some level of invasiveness, which may trigger stress responses in animals, potentially affecting their health and behavior [3,4,5,6]. In contrast, computer vision technology is a non-invasive method that uses cameras for individual identification, avoiding physical interference with animals and reducing the risk of injury and stress. It not only enables long-term and continuous tracking and analysis but also requires no direct contact with the animals, offering strong adaptability and better aligning with animal welfare requirements [7,8,9,10]. This technology relies on image recognition, providing an accurate, fast, and non-contact method of identification, with advantages such as low cost, non-invasive nature, and high efficiency [11,12]. The facial features of Hu sheep contain rich biological information, and there are significant differences between individuals, making facial recognition an effective means of identity verification. Additionally, this technology does not require physical contact with the animals, which helps avoid stress responses while ensuring efficient and accurate identification. Therefore, Hu sheep facial recognition technology is considered one of the most promising technologies in the field of identity recognition in the future [13,14,15,16].

In large-scale farming and sheep movement scenarios, individual animal recognition is a crucial task for improving management efficiency and optimizing production. In recent years, deep learning technology has made significant progress in the field of animal face recognition, especially in learning the biological features of sheep faces and individual identification. Many studies have employed different deep learning frameworks to improve the accuracy and speed of sheep face recognition [17,18]. However, despite the superior performance of existing deep learning methods (such as R-CNN and YOLO series) in object detection and face recognition, they still face several challenges in practical applications, primarily in terms of recognition accuracy, real-time performance, and adaptability. In animal face recognition research, the R-CNN series (such as Faster R-CNN) improves detection accuracy by using a two-stage detection structure (region proposal extraction and classification). Wang et al. proposed a Faster R-CNN-based method, which significantly enhanced the detection performance of dairy goats in surveillance videos through keyframe extraction, foreground segmentation, and region proposal modules, achieving an average accuracy of 92.49% [19]. However, this two-stage detection framework faces significant bottlenecks in real-time performance, with slower processing speeds that limit its application in large-scale farming environments. Unlike R-CNN, the YOLO (You Only Look Once) series models have gained widespread attention due to their single-stage detection framework and higher processing speed. Improved versions such as YOLOv5 can achieve real-time object detection through an end-to-end design. While YOLOv5s has achieved good results in face recognition, such as improving object detection accuracy by introducing multi-link convolution fusion blocks and re-parameterizable convolution structures, YOLO still exhibits limitations in handling small targets and complex backgrounds [20,21]. To address these challenges, researchers have attempted to combine other technologies to enhance the performance of YOLO models. For example, Wan et al. proposed a deep learning model based on the RepVGG algorithm and bilinear feature extraction fusion, which improves the YOLO model’s recognition capability at different poses and angles through attention mechanisms and enhanced feature extraction networks [22]. Additionally, Salama et al. optimized CNN parameters through data augmentation and Bayesian optimization, achieving 98% recognition accuracy, indicating that deep learning models still have great potential for application in small-scale datasets [23]. However, YOLO’s object detection accuracy decreases in complex backgrounds (such as cluttered environments and pose variations), especially in detecting small targets. Furthermore, while YOLO offers advantages in speed, its ability to adapt to high-density scenes is still limited, and it is prone to losing detailed information at lower resolutions. Therefore, finding a balance between real-time performance and accuracy remains a key challenge for current technologies.

Transfer learning, a novel technique that transfers knowledge from the source domain to the target domain, has shown great potential in animal recognition tasks. Zhang et al. proposed a sheep face recognition model, ViT-Sheep, based on Vision Transformers (ViTs), which improves recognition accuracy while effectively reducing training time and resource consumption by incorporating transfer learning, achieving a recognition accuracy of 97.9% [24]. Although this method has made significant progress in improving recognition accuracy, the ViT model still faces considerable challenges in training speed and computational resource consumption, especially in large-scale farming scenarios where real-time performance and computational efficiency are highly demanded. Additionally, Noor A et al. used the VGG16 model for sheep face image classification, achieving 100% training accuracy, 99.69% validation accuracy, and 100% test accuracy through transfer learning and data augmentation techniques. This indicates that the method has high accuracy in distinguishing normal and abnormal sheep faces and provides automated support for animal health monitoring [25]. However, the VGG16 model is prone to overfitting when faced with large datasets and, unlike YOLO, cannot achieve real-time recognition, which limits its scalability in practical applications.

Although deep learning models have made significant progress in the field of animal recognition, traditional manual labeling remains a key step in building high-quality datasets. However, manual labeling is not only time-consuming and labor-intensive but also prone to human errors, leading to inaccurate or inconsistent data labels, which creates several inconveniences for model training. In large-scale farming scenarios, with the continuous addition of new sheep, the cost and workload of manual labeling increase exponentially, significantly affecting the efficiency of model application [26,27,28].

To address the above-mentioned issues, this paper proposes an individual facial recognition method for Hu sheep based on automatic labeling and transfer learning. First, automatic labeling technology is employed to reduce the cost and time consumption of data labeling, especially when new sheep are introduced, significantly decreasing the workload of manual labeling and improving work efficiency. Second, this paper optimizes the YOLOv10n network structure, reducing the model’s computational consumption and storage requirements, thereby enhancing its real-time performance and deployability. Moreover, by integrating transfer learning, we can effectively transfer knowledge from the source domain to the small-sample target domain, facilitating the learning of new sheep characteristics, which in turn strengthens the model’s adaptability and generalization ability. Ultimately, this method significantly improves both the accuracy and efficiency of individual recognition tasks for Hu sheep.

The individual facial recognition method for Hu sheep based on automatic labeling and transfer learning proposed in this paper addresses the shortcomings of existing technologies, enabling efficient and accurate individual recognition in complex environments such as large-scale farming and sheep turnover. With continuous technological advancements, future research will focus on further optimizing model performance while exploring more flexible and scalable solutions to meet the growing demands of livestock management.

## 2. Materials and Methods

### 2.1. Data Collection

**Figure 1:**
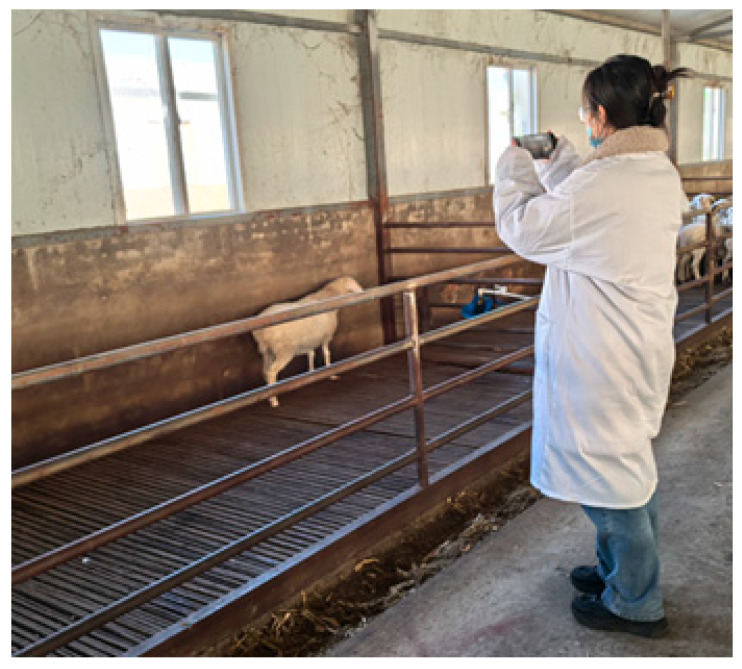
On-site shooting situation.

The Hu sheep face image data used in this study was sourced from Zhongsheng Huamei Sheep Farm in Xifeng County, Qingyang City, Gansu Province, with the data collection date in November 2024. The data was captured using video recording equipment with a resolution of 2272 × 1240 and a frame rate of 30 fps, creating a video dataset of Hu sheep faces covering various environments, postures, and angles. Video capture was conducted in an indoor farming area under natural lighting conditions. To comprehensively cover individual recognition of Hu sheep in different activity states, the data collection included various postures, such as standing, resting, walking, and grazing. Images were also captured from different angles, including front, side, and oblique views, as well as at varying heights. By encompassing these diverse environmental, posture, and angle conditions, the constructed dataset allows for a thorough evaluation of the sheep facial recognition system’s performance in various real-world scenarios. To avoid image quality degradation caused by the sheep’s stress response, the sheep were individually placed in a pen measuring 10 m in length and 3 m in width, allowing them to move freely during the experiment. The videographer followed the movements of the faces of the Hu sheep from outside the pen to record the video, as shown in Figure 1. To ensure the diversity and representativeness of the data, the video recordings of the Hu sheep in the pen were conducted at different angles, postures, and distances during two time periods: from 9:00 to 11:00 in the morning and from 2:00 to 5:00 in the afternoon. A total of 92 Hu sheep were included in this data collection, of which 82 sheep were used for training, with ear tag numbers ranging from s0 to s81. The remaining 10 sheep were used for transfer learning, with ear tag numbers ranging from t0 to t9. The age range of the sheep was 1 to 5 months, with a male-to-female ratio of 1:1. The video data for each Hu sheep had a duration of no less than one minute.

### 2.2. Data Preprocessing

**Figure 2:**
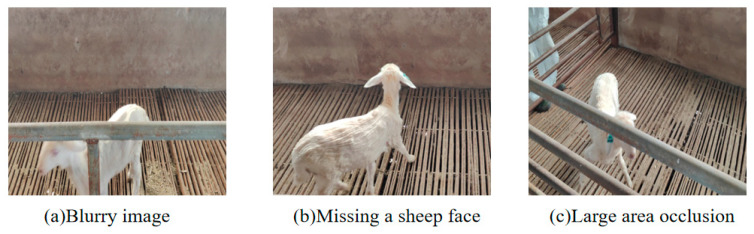
Examples of deleted images.

To ensure the quality of the Hu sheep face data, this study employed a multi-stage data cleaning process. First, based on the original video frame rate (30 fps), an interval sampling strategy was applied, extracting one image every 5 frames. This approach ensured that each video provided multiple image samples, thereby increasing the size of the dataset and covering different shooting angles and dynamic scene variations. Secondly, considering that fast movements and changes in lighting during video recording might cause images to be out of focus or blurry, a strict manual filtering process was applied. Images that were blurry and could not clearly identify the Hu sheep’s facial features, those with excessive occlusion of the sheep’s face, or those lacking the sheep’s face were deleted, as illustrated in Figure 2.

**Figure 3:**
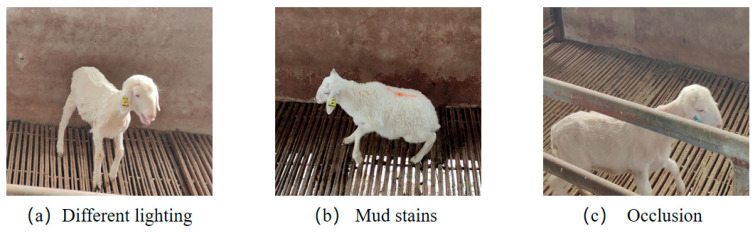
Different scenarios.

The background included in the filtered image is shown in Figure 3.

**Figure 4:**
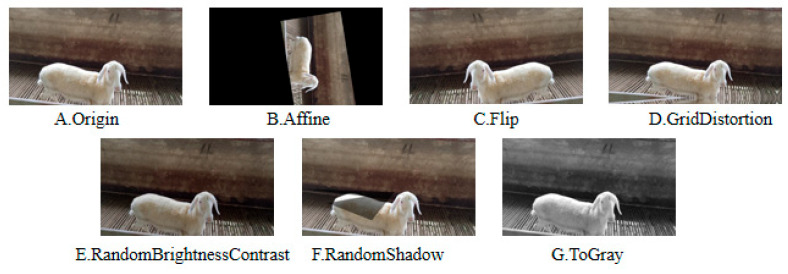
Comparison of augmented images.

In the research and application of image data augmentation, using various transformation strategies to enhance the original images has become an effective way to improve the performance of deep learning models. In this study, a probabilistically controlled hybrid augmentation strategy was adopted, and a hierarchical random augmentation pipeline was constructed using the Albumentations framework. This simulated the potential diversity of perspectives, environmental noise, image distortion, and dynamic occlusion that may occur in the complex scenarios of actual Hu sheep farming. The goal was to enhance the diversity of the Hu sheep face dataset and the model’s generalization ability. The specific augmentation methods included affine transformations, flipping, grid distortion, random brightness and contrast adjustments, adding shadows, and grayscale conversion. Figure 4 shows the effects of different data augmentation strategies.

**Figure 5:**
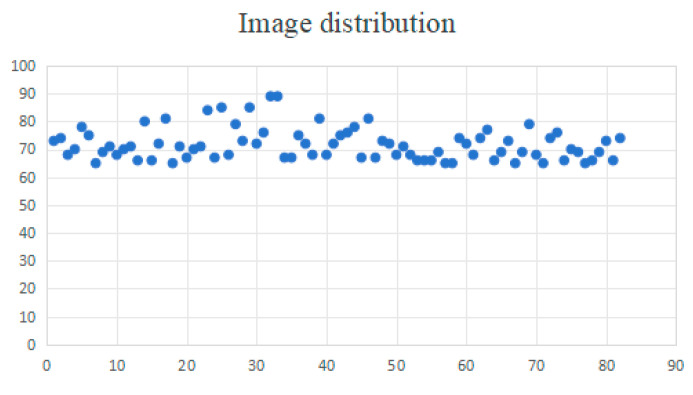
Image distribution.

After applying the six aforementioned data augmentation methods to 82 Hu sheep, a total of 6055 Hu sheep face images were obtained. The image distribution of 82 Hu sheep is shown in Figure 5 below:

After image augmentation, the dataset contains facial feature samples of each Hu sheep from different angles, postures, and positions. This diversified image augmentation method helps improve the model’s adaptability to different perspectives and environmental variations, ensuring that the facial features of each Hu sheep are fully learned. Although the number of images for each Hu sheep in the dataset is not perfectly consistent, it is generally balanced. The overall scale of the augmented dataset is sufficient to provide enough samples for feature learning, so the imbalance in data distribution will not significantly affect the learning process of the Hu sheep’s facial features.

### 2.3. Overall Framework

**Figure 6:**
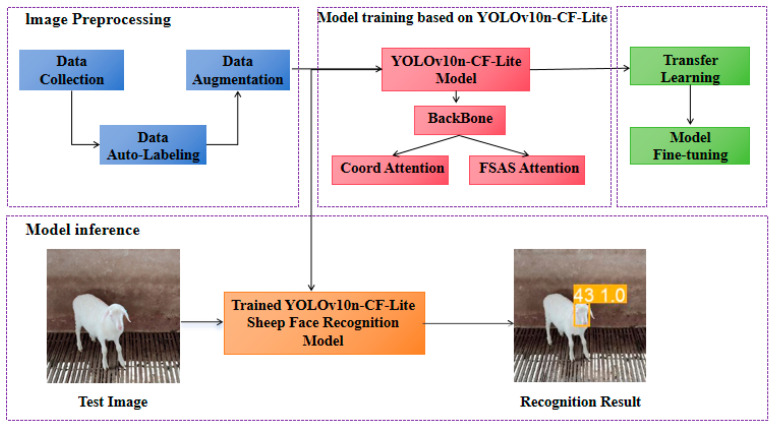
Overall flowchart.

This study proposes a Hu sheep individual face recognition method based on automatic labeling and transfer learning, with the overall workflow shown in Figure 6. To build a high-quality Hu sheep face recognition model, the research involved multi-angle video collection and data augmentation techniques to expand the dataset, ensuring that the model could obtain rich and representative data. An automatic labeling algorithm based on sheep face detection was designed, significantly reducing the labeling time cost. Through automatic labeling, manual intervention was minimized, and high-quality labeled data were provided for subsequent model training. Furthermore, a Hu sheep face recognition algorithm based on YOLOv10n-CF-Lite was proposed. Compared to traditional YOLO models, it offers higher computational efficiency and a lighter network structure, making it particularly suitable for complex farming environments. In addition, this study employed a transfer learning strategy to improve the model’s adaptability and generalization ability to newly introduced sheep. Furthermore, a Hu sheep face recognition algorithm based on YOLOv10n-CF-Lite was proposed. Compared to traditional YOLO models, it offers higher computational efficiency and a lighter network structure, making it particularly suitable for complex farming environments. In addition, this study employed a transfer learning strategy to improve the model’s adaptability and generalization ability to newly introduced sheep.

#### 2.3.1. Automatic Labeling

In the deep learning task of Hu sheep face recognition, precise bounding box annotations of sheep faces in each image are usually required. However, when the dataset is large, traditional manual annotation methods are not only time-consuming but also prone to inconsistency due to subjective biases from different annotators, especially when dealing with occlusions or blurred targets. To address these issues, this study proposes an automatic annotation method based on a pre-trained object detection model. Through iterative optimization, high-quality sheep face region labels can be generated. Furthermore, automatic annotation can conveniently assist farm managers in quickly identifying and collecting face data of newly introduced sheep, incorporating it into the database for subsequent individual management and tracking.

YOLOv10n, as a lightweight version of the YOLO series, possesses excellent object detection capabilities, enabling fast real-time detection with high precision, making it particularly suitable for applications in large-scale farming environments. Due to the frequent movement of sheep and the complexity of the environment, including variable lighting, cluttered backgrounds, and different postures, YOLOv10n demonstrates strong robustness and adaptability in these diverse settings. It can consistently recognize and label the sheep’s facial region, effectively reducing the impact of environmental factors on detection performance. Therefore, YOLOv10n not only meets the dual requirements of speed and accuracy for this research but also provides a reliable solution for automatic sheep face region labeling.

#### 2.3.2. YOLOv10n-CF-Lite Network Structure Design

**Figure 7:**
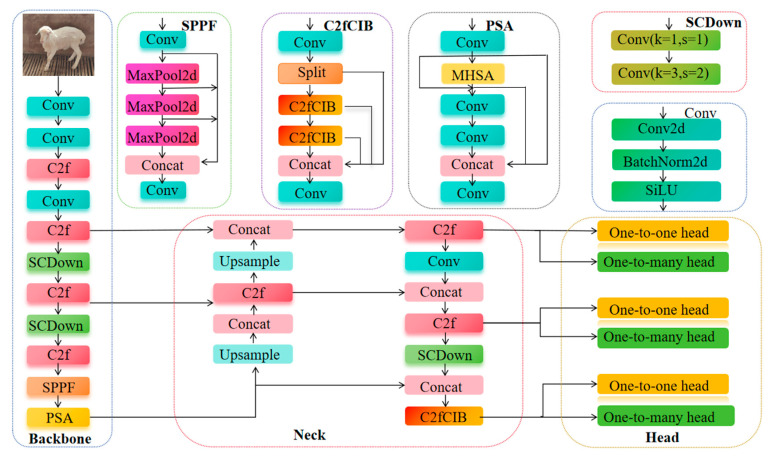
YOLOv10 network structure.

YOLOv10 is an efficient and lightweight object detection model, with its network structure shown in Figure 7. This model is an improvement based on YOLOv8, integrating depth-wise separable convolution, a lightweight network structure, feature fusion techniques, and an optimized detection head, aiming to provide fast and accurate object detection performance. The YOLOv10 network structure is primarily composed of three parts: Backbone, Head, and Neck. The Backbone extracts image features; the Neck performs multi-scale feature fusion through techniques such as feature pyramids; and the Head completes tasks such as classification, localization, and confidence prediction for object detection. The collaboration of these three components enables YOLOv10 to achieve efficient and precise object detection [29]. Considering the need for model efficiency and lightweight design in large-scale farming environments for Hu sheep individual recognition tasks, this study selects YOLOv10n as the baseline model to conduct research on Hu sheep face recognition.

**Figure 8:**
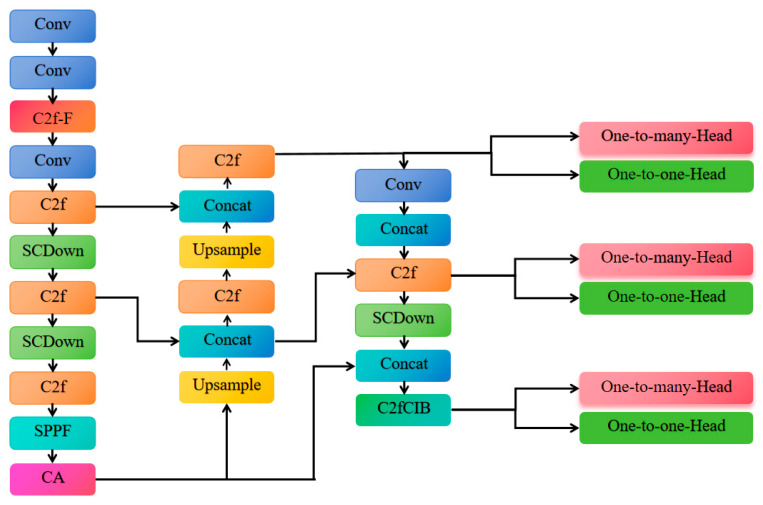
YOLOv10n-CF-Lite architecture diagram.

To achieve a balance between precision and efficiency in the task of Hu sheep face recognition, this study proposes the YOLOv10n-CF-Lite model, which is a lightweight improvement and feature-aware enhancement based on the YOLOv10n architecture. The network structure diagram is shown in Figure 8. The core improvements include a hierarchical attention fusion mechanism and a dual-path compression strategy. The YOLOv10n-CF-Lite model optimizes the network architecture and introduces an attention mechanism module, enhancing the model’s recognition precision while maintaining lightweight performance, particularly in terms of precision (P).

**Figure 9:**
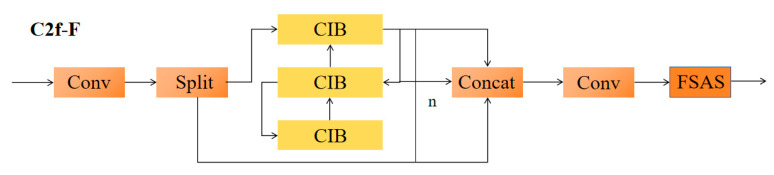
C2f-F architecture diagram.

The C2f-F architecture diagram is shown in Figure 9.

C2f-F is composed of two sequential convolutional layers (Conv-BN-SiLU) that extract local features and introduce Lightweight Self-Attention (LSA) to calculate global contextual dependencies.

(1) $$LSA(Q,K,V)=Soft\max\left(\frac{QK^{T}}{\sqrt{d_{k}}}\right)V$$

Here, Q, K, and V are generated from the input features through 1 × 1 convolutions, and d_k_ is the reduced number of channels.

The outputs of the main branch and cross-stage branch are dynamically weighted and fused through a Channel Attention Gate.

(2) $$F_{fused}=F_{main}⊗\sigma \left(f_{1\times 1}\left(F_{cross}\right)\right)+F_{cross}$$

Here, σ represents the Sigmoid function, and f_1×1_ represents the channel weight generator.

Residual connections are added to prevent gradient vanishing and retain the original feature distribution:

(3) $$Y=F_{fused}+X$$

##### Coordinate Attention

**Figure 10:**
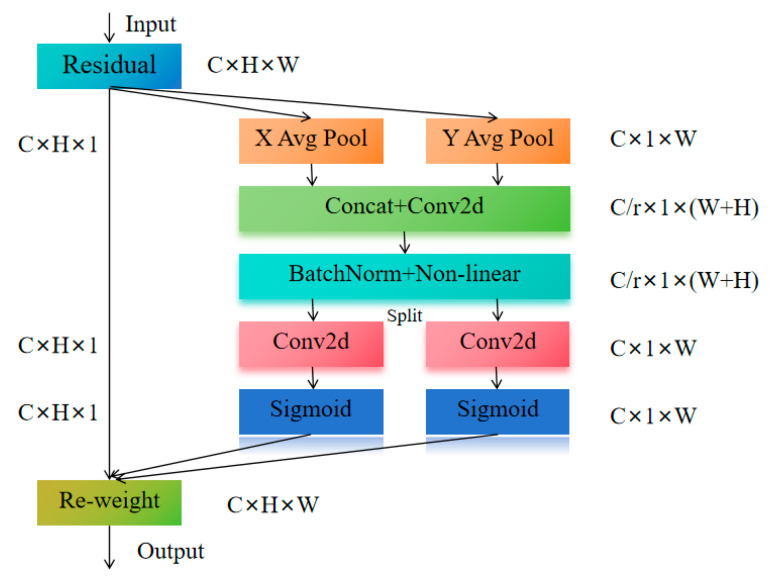
Coordinate Attention structure diagram.

The PSA module emphasizes features at different positions by calculating position-sensitive attention weights for each position. However, this position-based attention mechanism increases computational complexity, especially in high-resolution images, which may lead to a decrease in inference speed. Moreover, in some tasks, the PSA module’s overemphasis on position information may neglect other, more useful features, thus affecting model performance. To address the computational complexity issue of the PSA module in high-resolution image processing, we propose replacing PSA with Coordinate Attention (CA). CA explicitly incorporates spatial coordinate information and combines it with channel features for weighting, optimizing the model’s ability to perceive spatial structural information, as shown in Figure 10. Compared to PSA, Coordinate Attention can focus on important spatial location information in a more streamlined manner, thus enhancing the spatial response capability of the features. This optimization of spatial information is achieved with lower computational overhead, enabling the network to efficiently process high-resolution images while avoiding the additional computational burden introduced by PSA.

This mechanism models the coordinates in the horizontal and vertical directions of the feature map, allowing the network to focus more on key information in spatial locations. Specifically, Coordinate Attention optimizes the response capability of each position in the feature map by extracting positional information from each location and combining it with channel features. This enables the network to better capture features with significant discriminative power in spatial distribution. In the sheep face recognition task, the spatial structure of features is particularly important. Coordinate Attention optimizes the fusion of spatial location information and channel features, allowing the model to focus more on the key spatial features of the sheep’s face, rather than relying solely on positional sensitivity. Compared to PSA, Coordinate Attention not only enhances the model’s ability to perceive the spatial structure of the sheep’s face but also offers significant advantages in terms of computational complexity and inference speed, improving the model’s real-time performance and meeting the real-time application requirements in large-scale farming environments.

##### FSAS Attention

**Figure 11:**
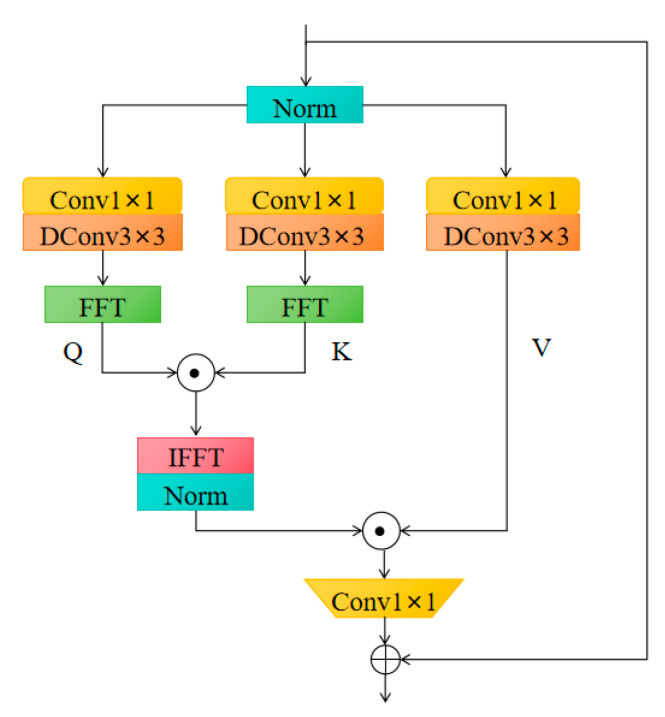
FSAS structure diagram.

FSAS (Frequency domain-based Self-Attention) is an attention module designed for lightweight object detection tasks, with its structure diagram shown in Figure 11. The core idea is to enhance the model’s attention to key regions through multi-scale feature selection and adaptive enhancement strategies, while suppressing irrelevant background interference.

The design of the FSAS module is based on a frequency-domain-based self-attention mechanism. Performing frequency-domain transformation on the feature maps enables the extraction and adaptive enhancement of multi-scale information. The input feature map is transformed from the spatial domain to the frequency domain. In the frequency domain, the different frequency components of the feature map reflect different spatial information levels in the image. Low-frequency components typically correspond to larger spatial structures, while high-frequency components represent detailed information and noise. In the frequency domain, self-attention is applied to the different frequency components. Self-attention calculates the relationships between each frequency component and others, dynamically selecting the most useful frequency components for the current task, thereby enhancing the model’s ability to perceive key areas. Compared to traditional spatial self-attention mechanisms, frequency-domain self-attention is better at focusing on the frequency information of the image, improving the ability to recognize details and structures. FSAS improves the model’s attention capabilities at different scales by introducing a multi-scale feature selection strategy. By adaptively selecting the appropriate scale and frequency components, FSAS can optimize the representation of feature maps at different resolutions, improving the overall recognition accuracy of the model. FSAS also suppresses the frequency information of irrelevant regions, reducing background noise interference in object recognition. In the frequency domain, the frequency components of the background area are usually more stable, and FSAS can effectively suppress these irrelevant components, thereby improving the model’s focus on the target.

This study enhances the model’s ability to focus on key features by embedding the FSAS attention mechanism after the CV2 convolution layer in the C2F module, thereby improving the model’s performance in real-world farming scenarios. The role of the C2F module is to increase feature diversity and expressive capability through cross-channel feature fusion, while FSAS further enhances the focus on critical regions from a frequency-domain perspective. The combination of both enables feature optimization across multiple scales and channels, and also improves the model’s local attention capability at the frequency-domain level. As a result, the model performs more stably and accurately in practical farming environments. Moreover, the FSAS module, through frequency-domain feature selection and enhancement, avoids the excessive consumption of computational resources typical of traditional attention mechanisms, maintaining a low computational complexity while improving the model’s ability to focus on key regions, making it suitable for lightweight object detection tasks.

#### 2.3.3. Transfer Learning Strategy

When the amount of data is limited, training a deep learning model from scratch often leads to overfitting, which restricts the model’s generalization ability and prevents it from effectively handling new samples. To address this issue, transfer learning, as an important technique, leverages the knowledge from pre-trained models and fine-tunes them, significantly improving training efficiency and model precision. Specifically, in livestock farms, when new sheep are introduced, facial data of the new individuals are typically collected and labeled, and then added to the existing database. This process accelerates the model update and training without starting from scratch. This approach not only effectively avoids overfitting but also greatly enhances the flexibility and efficiency of model updates. Particularly as the number of new sheep increases, it significantly improves the model’s maintainability and practical value. Transfer learning is a key method in machine learning, where the core idea is to transfer knowledge gained from a source task to a target task. It offers significant advantages, especially when target task data is scarce or training costs are high. In this study, transfer learning was successfully applied to facial recognition of Hu sheep. By fine-tuning a pre-trained model and incorporating data from specific objects and environments, it significantly improved the model’s performance on the target dataset. Compared to training a new model from scratch or using only the existing model, transfer learning not only effectively enhanced recognition precision but also substantially reduced training time.

In this study, the source dataset consists of 6055 images from 82 Hu sheep, while the target dataset contains 943 images from another 10 Hu sheep. These datasets share similar distributions and characteristics, and although the sample size is smaller, they still represent typical facial features of Hu sheep. Due to the limited sample size of the target dataset, training a model from scratch is prone to overfitting. Therefore, a transfer learning strategy was chosen to fully utilize the model weights trained on the source dataset to enhance the recognition performance on the target dataset.

Fine-tuning involves reusing the knowledge from a pre-trained model to optimize parameters on the target data, effectively addressing the issues of data scarcity and training efficiency. This method retains low-level features and adjusts the higher layers of the network to meet the specific task requirements. Compared to training from scratch, fine-tuning requires less data and converges more quickly. During the fine-tuning process, the learning rate is a critical hyperparameter, and both too high and too low rates can affect the training performance, making its proper setting crucial.

### 2.4. Experimental Parameter Settings

The experiments in this study were conducted in a Windows 11 environment, with the deep learning framework being PyTorch2.2.1, and training was performed using CUDA 12.1. The main experimental parameters are shown in Table 1.

### 2.5. Evaluation Metrics

In model evaluation, evaluation metrics are crucial for assessing the performance of a model. For the Hu sheep face recognition task, the evaluation metrics should not only consider precision but also focus on real-time performance and resource consumption in practical applications. Therefore, this study adopts multiple evaluation metrics to comprehensively assess the performance of the Hu sheep face recognition model based on automatic annotation. The primary evaluation metrics used in this study are precision, recall, F1 score, model parameters, IOU, and memory size.

Precision refers to the proportion of samples predicted as Hu sheep faces that are actually Hu sheep faces. The equation for calculating precision is as follows:

(4) $$Precision=\frac{TP}{TP+FP}$$

The higher the precision, the fewer false positives the model has when recognizing Hu sheep faces.

Recall measures how many true Hu sheep faces the model can successfully identify. A high recall means the model reduces the occurrence of missed detections, ensuring that most Hu sheep faces are correctly recognized. The equation for calculating recall is as follows:

(5) $$Recall=\frac{TP}{TP+FN}$$

where TP represents true positives, TN represents true negatives, FP represents false positives, and FN represents false negatives.

The F1 score is the harmonic mean of precision and recall, which comprehensively considers the model’s precision and recall ability. The equation for calculating the F1 score is as follows:

(6) $$F_{1}=\frac{2\times Precision\times Recall}{Precision+Recall}$$

A higher F1 score means the model can effectively improve recall while maintaining high precision, thus reducing the risks of false positives and false negatives.

Mean Average Precision (mAP) reflects the model’s precision and recall when detecting Hu sheep faces, and it helps us understand the model’s performance under different detection thresholds. The equation for calculating mAP is as follows:

(7) $$mAP=\frac{1}{N}\sum_{c=1}^{N}AP_{C}$$

where N represents the number of target categories, and AP_C_ represents the average precision of the c-th category. In this study, mAP can effectively assess the YOLOv10 model’s ability to detect Hu sheep faces, especially in the model after automatic labeling and lightweight improvements.

In addition to the traditional precision metrics mentioned above, considering YOLOv10’s lightweight design and practical application scenarios, this study also focuses on the model’s size. Therefore, we adopted the following metrics related to computational resources:

Model Parameters: This metric measures the complexity of the model. Generally, the fewer the parameters, the lighter the model, making it suitable for running in resource-constrained environments such as embedded devices.

Memory Usage: This metric represents the amount of memory required by the model during inference. Lower memory usage helps the model run on low-power devices.

IOU (Intersection over Union) is a commonly used evaluation metric in object detection, which is used to measure the overlap between the predicted box and the ground truth box. To calculate IOU, it is necessary to convert the center coordinates and dimensions given in YOLO format to the top-left, Equation (8), and bottom-right coordinates, Equation (9):

(8) $$\left(x_{2},y_{2}\right)=\left(\frac{x\_center+width}{2},\frac{y\_center+heigeh}{2}\right)$$

(9) $$\left(x_{1},y_{1}\right)=\left(\frac{x\_center-width}{2},\frac{y\_center-height}{2}\right)$$

(10) $$IoU=\frac{IntersectionArea}{UnionArea}$$

IntersectionArea refers to the area of the overlapping region between the two boxes, while UnionArea refers to the sum of the areas of the two boxes minus the area of their overlap.

Through the above multidimensional evaluation metrics, we can gain a comprehensive understanding of the performance of the Hu sheep face recognition model based on automatic labeling and the lightweight YOLOv10, thus providing a scientific basis for subsequent model optimization and deployment.

## 3. Experiment and Results

### 3.1. Auto-Labeling Results

In this study, the best weights (best.pt) obtained by training YOLOv10n on the initial manually labeled dataset (500 images) were used as the initialization parameters for the automatic labeling model. The model retained the general feature extraction capability of the COCO pre-trained Backbone network, with a fixed input image size of 640 × 640 pixels. For each input image, the model generates a text file with the same name (e.g., IMG_001.jpg corresponds to IMG_001.txt). The content of the file includes the sheep face category name, the coordinates of the bounding box center, and the width and height of the bounding box.

In this paper, for the automatic annotation task of sheep face regions, the sensitivity of the T (threshold) parameter in the MAD (Mean Absolute Deviation) component to annotation performance was analyzed. By conducting experiments with three different thresholds, T = 1.0, T = 1.5, and T = 2.0, the performance variations in the model under different T values were observed, as shown in Table 2.

The experimental results indicate that the T value has a bidirectional effect on the model’s performance. A lower T value (e.g., T = 1.0) allows the model to accept more labeled areas with greater leniency, improving both precision and recall. However, this may lead to some mislabels or redundant annotations, affecting the overall performance (mAP@0.5). As the T value increases, the model becomes stricter, resulting in a slight improvement in precision but a slight decrease in recall. At T = 1.5, the model’s overall performance is balanced, with both precision and recall remaining stable. At T = 2.0, although recall decreases, both precision and mAP@0.5 slightly improve, suggesting that higher T values may help reduce mislabels but could also lead to some missed labels.

By analyzing the impact of different T values on labeling performance, the conclusion is drawn that selecting an appropriate T value is crucial for the performance of the sheep face region auto-labeling task. A lower T value is suitable for tasks that prioritize higher recall and precision, while a higher T value helps improve precision and reduce mislabels, but may sacrifice recall. Considering that the focus of this study is on optimizing precision and reducing redundant annotations, T = 2.0 is selected as the final threshold, as it effectively reduces mislabels while maintaining a balanced overall performance.

**Figure 12:**
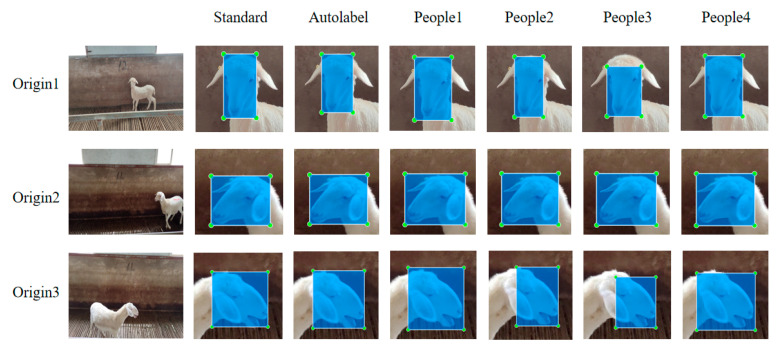
Auto-labeling comparison image.

To verify the effectiveness of the proposed automatic labeling model, a comparative experiment was designed. In the experiment, a standard labeled dataset was selected, and the results of automatic labeling and four manual annotators were compared with the standard labeled results. The comparison of the sheep face region annotations is shown in Figure 12.

The similarity between the automatic labeling results, manual labeling results, and the ground truth annotations is evaluated by calculating the Intersection over Union (IoU). The IoU comparison results are shown in Table 3.

The experimental results show that the IoU values of the automatic labeling are generally higher than those of the four manual annotators, and in most cases, the automatic labeling results not only approach the standard labeling but, in some instances, even surpass manual annotations. This result fully validates the significant advantages of the proposed automatic labeling method in terms of labeling precision and efficiency, achieving results comparable to or even better than manual labeling. Therefore, it is evident that this method has good feasibility for sheep individual recognition tasks, effectively reducing the reliance on manual labeling and providing an efficient and reliable solution for labeling large-scale datasets.

**Figure 13:**
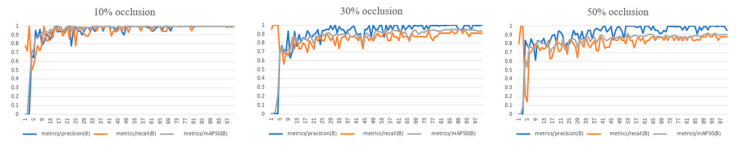
Different levels of occlusion.

To analyze the impact of occlusion levels on automatic labeling performance, we conducted experiments on images of sheep face regions with different occlusion levels (10%, 30%, and 50%) and evaluated the accuracy and recall of automatic labeling, as shown in Figure 13.

The experimental results indicate that under lower occlusion conditions, the labeling accuracy remains at a high level, but as the occlusion ratio increases, the labeling performance declines. Particularly under 50% occlusion, there is a significant drop in labeling accuracy, suggesting that occlusion has a significant impact on labeling performance. We believe that occlusion may lead to the loss of key facial features, and therefore, future research will further explore how to improve the model’s robustness to occlusion by enhancing labeling algorithms or adding training data with simulated occlusion.

To further ensure the credibility of the automatic labeling results and perform error correction, this study established a manual sampling verification and correction mechanism and used the open-source tool LabelImg for pixel-level correction of erroneous labels. Through verification, it was found that the automatic labeling errors were primarily concentrated in three categories, localization bias, missed detections, and false detections, with an error occurrence probability of 1%, indicating that the automatic labeling of image data has a high level of credibility.

The automatic labeling method proposed in this paper significantly reduces labor costs while ensuring labeling quality (*p* ≥ 0.9), decreasing the time required per image from 0.067 min to 0.014 min, representing a 79.1% reduction in time consumption. This provides a feasible solution for large-scale agricultural data labeling projects.

### 3.2. Ablation Experiment

In order to further validate the effectiveness of each attention mechanism proposed in this study, this section systematically evaluates the impact of different modules on the final performance through ablation experiments. By progressively adding or replacing certain design elements in the model, we are able to deeply analyze the contribution of each factor to the overall performance of the model.

The experiments in this section used 6055 images of 82 heads of Hu sheep, which were divided into training and validation sets in a ratio of 8:2. The four models in Table 4 include different combinations of two improvement strategies. The same training parameters were used for each model, with model E0 serving as the baseline without any improvements, and EP containing all the improvement strategies.

The experimental results show that by improving the model structure and replacing the original PSA module with the Coordinate Attention mechanism module (E1), the model’s precision in Hu sheep face recognition increased by 0.4%. The model’s parameter count decreased by 0.24 M, and its size reduced by 0.5 MB. This demonstrates that the Coordinate Attention mechanism module significantly reduces computational complexity while maintaining recognition performance, making a significant contribution to the model’s lightweight performance. The FSAS attention mechanism module plays a key role in improving the model’s Hu sheep face recognition precision. After adding this module (E2), the recognition precision increased by 2.1%, and mAP@0.5 improved by 0.5%. At the same time, the model’s parameter count increased by 0.01 M. This indicates that the FSAS module can effectively capture key channel features of sheep faces, significantly enhancing individual distinguishability. Although the FSAS module greatly contributes to improving Hu sheep face recognition precision, it compromises the model’s lightweight performance, leaving significant room for optimization. To balance the precision and lightweight requirements of the Hu sheep individual recognition task, we designed the CA-FSAS hybrid attention architecture (EP). The CA module is used at the end of the Backbone to replace the original PSA module, compressing redundant features, while the FSAS module is embedded after the CV2 convolution in the C2F module. The results show that the final model’s recognition precision increased by 2.2%, mAP@0.5 improved by 0.7%, the model’s parameter count decreased by 0.23 M, and the model size was reduced by 0.5 MB. This indicates that the YOLOv10n-CF-Lite (EP) model effectively meets the dual demands of Hu sheep face recognition precision and lightweight performance.

### 3.3. Model Comparison

To verify the effectiveness of the proposed YOLOv10n-CF-Lite model, we conducted a comparative evaluation of YOLO series lightweight models, Faster R-CNN, and SSD on the Hu sheep individual recognition task. For this purpose, multiple comparative experiments (Table 5) were designed, and the models were comprehensively evaluated using metrics such as precision, recall, mAP@0.5, F1 score, and model size.

The face recognition precision of the Hu sheep using YOLOv10n-CF-Lite reached 92.3%, and the mAP@0.5 achieved 96.2%. Compared to YOLOv6n [30], YOLOv8n, YOLOv10n, YOLOv11, Faster R-CNN [31], and SSD [32], the precision was higher by 26.3%, 38.5%, 2.2%, 0.7%, 4.8%, and 9%, respectively; mAP@0.5 was higher by 16.4%, 33.5%, 0.7%, 1%, 6.5%, and 11.2%, respectively. These results indicate that YOLOv10n-CF-Lite has stronger precision in Hu sheep individual recognition, particularly excelling in complex backgrounds and occluded scenarios. In terms of lightweight model performance, YOLOv10n-CF-Lite reduces the number of parameters compared to YOLOv6n, YOLOv8n, YOLOv11, Faster R-CNN, and SSD by 1.67 M, 0.63 M, 0.08 M, 39.23 M, and 22.06 M, respectively. The model size is reduced by 3.2 MB, 1.2 MB, 0.1 MB, 153.9 MB, and 57.3 MB, respectively. The experimental results show that YOLOv10n-CF-Lite maintains high recognition precision while effectively achieving model lightweighting in the Hu sheep individual recognition task, fully meeting practical application needs. FPS (Frames Per Second) is an important metric for evaluating model inference efficiency. Compared to traditional Faster R-CNN (44.7 FPS), SSD (80.6 FPS), YOLOv6n (625 FPS), and YOLOv8n (192 FPS), the inference speed of YOLOv10n-CF-Lite is significantly superior, demonstrating its ability to achieve efficient inference performance even in complex tasks. In comparison to YOLOv10n (714 FPS) and YOLOv11 (714 FPS), YOLOv10n-CF-Lite not only maintains inference efficiency but also further optimizes computational performance, making it more suitable for real-time demanding scenarios in practical applications. It shows great potential for individual identification tasks in sheep face recognition, offering significant advantages.

### 3.4. Transfer Learning Performance Verification

To verify the transfer learning performance of the YOLOv10n-CF-Lite model in the Hu sheep individual recognition task, this section provides a detailed analysis of the transfer learning experiment results. The experiment involves 10 new individual Hu sheep from the target dataset, and compares the performance of YOLOv10n, YOLOv10n-CF-Lite, and a YOLOv10n-CF-Lite model pre-trained with weights from the source dataset. In the experiment (Table 6), the training learning rate for all models is set to lr0 = 0.01, with lrf = 1.0 explicitly set and the learning rate scheduler disabled to ensure the learning rate remains constant during training.

**Figure 14:**
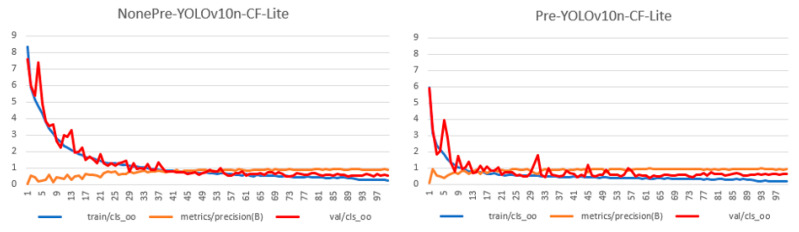
Comparison of transfer learning parameters.

As shown in the table, after transfer learning, the YOLOv10n-CF-Lite model achieved a recognition precision of 92.7%, mAP@0.5 of 95.2%, and an F1 score of 91.0% on the target dataset, which represents a significant improvement in Hu sheep face recognition precision compared to the model without pre-training. This indicates that, despite the relatively small size of the target dataset, transfer learning can effectively help the model adapt to new data and significantly accelerate the fitting speed of features during training, as shown in Figure 14. When the best weights from the source dataset were not used for pre-training, YOLOv10n-CF-Lite required 43 epochs of training to achieve relatively good performance. However, when transfer learning was applied to the target dataset, precision exceeded 90% after just 14 epochs of training, significantly improving the model’s convergence speed. Furthermore, the transfer learning model’s validation loss decreased more rapidly and maintained lower losses on both the training and validation sets, demonstrating better generalization ability.

The results of the transfer learning experiment show that the Hu sheep face recognition model based on automatic labeling and the lightweight YOLOv10 (YOLOv10n-CF-Lite) can effectively transfer knowledge from the source dataset to the target dataset. It significantly shortens training time, enhances model adaptability, and maintains high precision and inference speed. In the Hu sheep individual recognition task, when new sheep are introduced to the farm, the manager can quickly incorporate them into the database and accelerate model training by collecting and labeling new sheep face data, without the need to start training from scratch, thus avoiding inefficient use of time. Therefore, transfer learning demonstrates its significant application value in situations with data scarcity and the need for efficient management.

## 4. Discussion

### 4.1. Comparison with Other YOLO Versions

To verify the superiority of YOLOv10n-CF-Lite in the Hu sheep individual recognition task, a comprehensive comparison was made with other versions of the YOLO series, as shown in Table 7. Among all versions, YOLOv10n-CF-Lite outperformed YOLOv5, YOLOv8, and YOLOv10 in both precision and mAP@0.5. For example, YOLOv10n-CF-Lite’s precision was 20.2%, 15.1%, and 23.8% higher than YOLOv5s, YOLOv8s, and YOLOv10s, respectively; its mAP@0.5 was 1.9%, 1.8%, and 7.2% higher than YOLOv5l, YOLOv8l, and YOLOv10x, respectively.

YOLOv10n-CF-Lite outperforms other versions in both model size and parameters, two key lightweight metrics. For example, YOLOv10n-CF-Lite’s model size is reduced by 39.3 MB, 41.5 MB, and 28.2 MB compared to YOLOv5m, YOLOv8m, and YOLOv10m, respectively. The number of model parameters is reduced by 5.31 M, 7.32 M, and 5.56 M compared to YOLOv5s, YOLOv8s, and YOLOv10s, respectively. YOLOv10n-CF-Lite outperforms all other YOLO series models in terms of FPS. Although YOLO series models generally offer high inference speeds, YOLOv10n-CF-Lite further enhances inference performance by optimizing the model architecture and computational processes, making it more competitive in real-time inference tasks. At the same time, it maintains a good balance in precision, ensuring efficient inference and high detection accuracy. This makes it an ideal solution for real-time demanding scenarios in practical applications.

### 4.2. Shortcomings

Although this study has achieved certain results in the Hu sheep face recognition task, several limitations still exist. For example, the current dataset is primarily collected from Hu sheep in the Gansu region, and the generalization ability for high-altitude breeds (such as Tibetan sheep) has not been verified. Additionally, there is insufficient data coverage under extreme weather conditions (such as heavy rain and sandstorms), which limits the model’s adaptability and robustness in complex environments. The current transfer learning experiments are limited to within-breed transfer (Hu sheep → Hu sheep), and the model’s performance in cross-breed transfer (e.g., Sunite sheep → Hu sheep) is unknown. The model’s ability to learn cross-domain features requires further enhancement. Moreover, the threshold for the target domain data volume has not been clarified. When the target domain sample size is less than 500 images, it remains to be explored whether the model will experience negative transfer after fine-tuning. Based on the above analysis, future research can be conducted in the following directions: First, the data collection range can be expanded to cover more regions and sheep breeds under different climatic conditions (such as heavy rain and sandstorms), in order to enhance the model’s adaptability and generalization ability in diverse environments. Second, a deeper study of the feature differences between different sheep breeds (such as Hu sheep and Sunite sheep) can be carried out, and more flexible and effective transfer learning strategies can be designed to explore how to achieve effective transfer of knowledge across breeds. Third, research can focus on how to detect negative transfer through intelligent methods, enabling timely identification and prevention of negative transfer during the migration process. Finally, exploring adaptive fine-tuning strategies, which dynamically adjust model parameters to overcome performance degradation caused by insufficient data, will further enhance the model’s stability and generalization ability.

### 4.3. Model Fine-Tuning Learning Rate

For the small sample cross-domain adaptation problem in Hu sheep individual recognition, traditional global learning rate strategies tend to lead to model overfitting or underfitting. This study employs a model fine-tuning learning rate method, which controls fine-grained parameter updates to enable effective transfer of cross-domain features. Based on transfer learning theory [33], when the target domain has a small amount of data (N = 10) and there is a distribution discrepancy between the source and target domains (e.g., Hu sheep of different age groups), a higher learning rate (0.001–0.01) helps accelerate the model’s adaptation to the new task while avoiding excessive disturbance of low-level features. This range selection is based on the fine-tuning experience of ImageNet pre-trained models [34]. Within this interval, the learning rate (lr0) is discretely sampled with a step size of 0.001, and training and validation are conducted at each learning rate to find the optimal one, ensuring that the model achieves the best recognition performance on the target dataset. The experimental results of the fine-tuning learning rate are shown in Table 8.

By comparing the model precision and convergence speed at different learning rates, we found that a learning rate of 0.007 achieved the best performance on the target dataset. At this learning rate, the model converged more quickly and showed better precision and F1 score values on the target dataset compared to other learning rate settings. Based on this experimental result, we chose a learning rate of 0.007 as the optimal rate for the fine-tuning phase of transfer learning in this study on Hu sheep.

### 4.4. Cross-Breed Transfer

Significant progress has been made in transfer learning for individual identification tasks within the same breed (Hu sheep). However, cross-breed transfer learning faces greater challenges, as differences in data distribution and biological characteristics may lead to a decline in model performance. Therefore, cross-breed transfer learning has become a pressing research issue. This paper experimentally validates the application of transfer learning between different sheep breeds, specifically exploring the feasibility and effectiveness of transfer learning between Hu sheep (source breed) and Sunite sheep (target breed). We used the YOLOv10n-CF-Lite model to transfer the pre-trained model from the Hu sheep dataset (82 sheep, 6055 images) to the Sunite sheep dataset (15 sheep, 960 images) in order to test the impact of cross-breed transfer learning on sheep individual identification precision. The experimental results are shown in Table 9.

As shown in the table, by transferring the pre-trained model from the Hu sheep dataset to the Sunite sheep dataset, we observed a significant performance improvement. Specifically, the transferred model achieved precision, mAP@0.5, and F1 score increases of 20.1%, 18.7%, and 17.6%, respectively, on the Sunite sheep dataset. YOLOv10n-CF-Lite shows a significant improvement in FPS compared to the YOLOv10n model. Although there is a slight increase in FLOPS, the enhanced inference efficiency means the model performs excellently in efficient inference tasks. In transfer learning, while pre-trained weights help accelerate model convergence, they may also slightly decrease FPS due to the additional fine-tuning and adaptation process. However, the strategy of using pre-trained weights still maintains the advantage of relatively low computational complexity and contributes to achieving good performance in new tasks. The experimental results indicate that cross-breed transfer learning performs significantly better on the Sunite sheep compared to models trained from scratch solely on Sunite sheep data. Cross-breed transfer learning effectively helps the model extract general features from the source breed’s knowledge, thus improving the recognition accuracy of the target breed. This also provides effective support for further enhancing the model’s generalization ability and task adaptability.

According to Table 6 and Table 9, although pre-trained models were used, the results of transfer learning on Sunite sheep were still inferior to the transfer results on the Hu sheep breed. This suggests that the model may not have fully adapted to the characteristics of Sunite sheep. Although both Hu sheep and Sunite sheep are sheep breeds, there are certain differences in facial features, such as fur type, facial structure, and other physiological characteristics, which can affect the transfer learning performance of facial recognition models. Additionally, the effectiveness of transfer learning is influenced by the training dataset. When using YOLOv10n to train on Sunite sheep data, although the accuracy improved, the results were still not as expected compared to training on the Hu sheep breed. This may be because the Sunite sheep dataset is smaller and less diverse compared to the Hu sheep training dataset, which leads to the model being unable to capture sufficient features. The improved YOLOv10n model shows some improvement, but due to the inter-breed feature differences, it still cannot match the performance achieved when transferring from the Hu sheep breed. To address this issue, we plan to explore further improvement methods in future work. For example, increasing the diversity of Sunite sheep data through data augmentation techniques or narrowing the feature gap between source and target breeds using domain adaptation techniques. Additionally, methods like multi-task learning or Generative Adversarial Networks (GANs) may help enhance the model’s adaptability during cross-breed transfer learning.

## 5. Conclusions

This study proposes a technical framework that integrates data augmentation, automated labeling, and lightweight model optimization for the individual identification task of Hu sheep. The experimental results show that this framework significantly improves recognition precision and reduces computational costs. By collecting Hu sheep video data under various distances, multiple angles (front/side/top view), and dynamic occlusion scenarios, a dataset containing 6055 high-quality images was constructed. Compared to traditional manual labeling, the automated labeling system based on YOLOv10n improved labeling efficiency by 79.1%, while maintaining high labeling consistency. A CA-FSAS hybrid attention architecture is designed based on YOLOv10n, enhancing the model’s ability to perceive the spatial structure of sheep faces through a feature recalibration strategy guided by coordinate information. The experimental results show that after optimization, the recognition precision of Hu sheep faces increased to 92.3% and mAP@0.5 improved to 96.2%, while the model’s parameter size was reduced by 8.4%, achieving effective model lightweighting. Additionally, transfer learning was applied to transfer the model parameters trained on the source domain (82 Hu sheep with 6055 images) to the target domain (10 new individuals with 943 images). By fine-tuning the learning rate of the model, it was found that the best recognition performance occurred when the learning rate was 0.007. At this point, the recognition precision on the target domain test set increased by 3.3%, and mAP@0.5 improved by 0.7%.

Overall, this study significantly improved the precision and efficiency of individual identification tasks for Hu sheep through automated labeling, YOLOv10 improvements, lightweighting, and the application of transfer learning, providing a practical solution for real-world applications. However, there are still some limitations, and future research will focus on enhancing dataset expansion, model generalization, and further optimization of robustness, aiming to drive continuous development in this field.

## Figures and Tables

**Table 1:** Experimental parameters.

Config	Value
Processor	AMD Ryzen 9 7950X
RAM	128 GB
Graphics	NVIDIA RTX A5000 × 2
Optimizer	SGD
Batch size	16
Training epochs	100
Image size	640 × 640

**Table 2:** T value setting.

T	Precision/%	Recall/%	mAP@0.5/%
1.0	96.5	99.8	99.1
1.5	93.4	98.2	99.0
2.0	99.0	98.2	99.3

**Table 3:** IoU results.

	Auto-label	People1	People2	People3	People4
Origin1	0.9682	0.8204	0.8807	0.7414	0.8510
Origin2	0.9499	0.9045	0.9144	0.8798	0.9259
Origin3	0.9216	0.8827	0.7702	0.6216	0.8241

**Table 4:** Ablation experiment results.

Model	CA Replace PSA	FSAS	Precision /%	Recall /%	mAP@0.5 /%	mAP@0.9 /%	F1 /%	Model Size /MB	Parameters /M
E0			90.1	89.1	95.5	81.7	89.6	5.9	2.77
E1	√		90.5	88.7	95.2	81.2	89.6	5.4	2.53
E2		√	92.2	87.4	96.0	82.6	89.7	5.9	2.78
EP	√	√	92.3	89.1	96.2	82.4	90.7	5.4	2.54

**Table 5:** Model comparison results.

	Precision /%	Recall /%	mAP@0.5 /%	F1 /%	Model Size /MB	Parameters /M	FPS /Batch Size = 16
YOLOv6n	66	74.3	79.8	69.9	8.6	4.21	625
YOLOv8n	53.8	57.1	62.7	55.4	6.6	3.17	192
YOLOv10n	90.1	89.1	95.5	89.6	5.9	2.77	714
YOLOv11	91.6	87.5	95.2	89.5	5.5	2.62	714
Faster R-CNN	87.5	80.9	89.7	84.1	159.3	41.77	44.7
SSD	83.3	78.6	87.0	80.9	62.7	24.6	80.6
YOLOv10n-CF-Lite	92.3	89.1	96.2	90.7	5.4	2.54	667

**Table 6:** Transfer learning results.

	Precision /%	Recall /%	mAP@0.5 /%	mAP@0.9 /%	F1 /%	Model Size /MB	Parameters /M
YOLOv10n	90.8	89.9	94.5	84.6	90.3	5.8	2.70
YOLOv10n-CF-Lite	91.2	89.5	96.3	86.1	90.3	5.3	2.47
Pre-YOLOv10n-CF-Lite	92.7	89.3	95.2	87.2	91.0	5.3	2.47

**Table 7:** Comparison of YOLO series versions.

	Precision /%	Recall /%	mAP@0.5 /%	F1 /%	Model Size /MB	Parameters /M	FPS /Batch Size = 16
Yolov5s	72.1	75.9	82.4	73.6	16.0	7.85	500
Yolov5m	89.5	83.9	92.8	86.6	44.7	22.16	313
Yolov5l	88.8	88.0	94.3	88.4	96.5	47.98	204
Yolov5x	90.8	86.7	94.4	88.7	178.8	89.07	125
Yolov8s	77.2	83.8	87.4	80.4	20.0	9.86	167
Yolov8m	90.3	87.0	94.2	88.6	46.9	23.25	114
Yolov8l	89.7	88.0	94.4	88.8	79.4	39.56	85
Yolov8x	92.2	90.9	96.2	91.5	123.8	61.67	67
Yolov10m	77.2	80.6	88.4	78.9	33.6	16.55	164
Yolov10s	68.5	67.3	76.3	67.9	16.6	8.10	182
Yolov10x	77.7	82.6	89.0	80.1	64.4	31.74	122
YOLOv10n-CF-Lite	92.3	89.1	96.2	90.7	5.4	2.54	667

**Table 8:** Learning rate comparison.

Learning Rate	Precision /%	Recall /%	mAP@0.5 /%	mAP@0.9 /%	F1 /%	Training Time/h	Convergence Epochs
0.001	91.9	90.8	96.3	86.7	91.3	0.209	29
0.002	92.1	92.6	97.0	87.1	92.3	0.207	20
0.003	89.1	92.0	96.4	86.9	90.5	0.206	15
0.004	95.0	91.4	96.0	87.2	92.9	0.206	14
0.005	93.4	92.5	97.2	87.2	92.9	0.208	14
0.006	94.8	90.9	96.0	86.8	92.8	0.208	14
0.007	94.9	92.4	97.0	87.5	93.6	0.208	14
0.008	91.1	93.5	96.7	87.2	92.3	0.208	14
0.009	91.9	91.1	96.8	88.0	91.5	0.208	14
0.01	92.7	89.3	95.2	87.2	91.0	0.346	14

**Table 9:** Cross-breed transfer results.

	Precision /%	Recall /%	mAP@0.5 /%	F1 /%	Model Size /MB	Parameters /M	FLOPS /G	FPS /Batch Size = 16
YOLOv10n	57.2	65.1	68.1	60.9	5.8	2.70	8.3	345
YOLOv10n-CF-Lite	56.8	63.8	68.4	60.1	5.3	2.47	8.4	370
Pre-YOLOv10n-CF-Lite	76.9	78.5	87.1	77.7	5.3	2.47	8.4	323

## Data Availability

All datasets during the current study are available from the corresponding author upon reasonable request.
